# Stilbene Treatment Reduces Stemness Features in Human Lung Adenocarcinoma Model

**DOI:** 10.3390/ijms251910390

**Published:** 2024-09-27

**Authors:** Vittoria Livraghi, Alice Grossi, Anna Scopelliti, Giorgia Senise, Luciano Augusto Gamboa, Samantha Solito, Lucia Anna Stivala, Virginie Sottile, Monica Savio

**Affiliations:** 1 Department of Molecular Medicine, Immunology and General Pathology Unit, University of Pavia, 27100 Pavia, Italy; vittoria.livraghi01@universitadipavia.it (V.L.); alice.grossi02@universitadipavia.it (A.G.); anna.scopelliti01@universitadipavia.it (A.S.); giorgia.senise01@universitadipavia.it (G.S.); lucianoaugusto.gamboa01@universitadipavia.it (L.A.G.); luciaanna.stivala@unipv.it (L.A.S.); 2 Centro Grandi Strumenti (CGS), University of Pavia, 27100 Pavia, Italy; samantha.solito@unipv.it

**Keywords:** A549 cells, resveratrol, 4,4’-dihydroxy-trans-stilbene, stemness markers

## Abstract

Lung cancer is among the most clinically challenging tumors because of its aggressive proliferation, metastasis, and the presence of cancer stem cells (CSCs). Natural bioactive substances have been used for cancer prevention, and, in particular, resveratrol (RSV), a stilbene-based compound with wide biological properties, has been proposed for chemoprevention. Its lesser-known analogue 4,4’-dihydroxy-trans-stilbene (DHS) has demonstrated superior activity both in cell-based assays and in mouse and zebrafish in vivo models. The present study analyzed the effects of DHS and RSV on A549 lung cancer cells, with a particular focus on stemness features and CSCs, isolated by sorting of the side population (SP). The results show that both stilbenes, especially DHS, strongly inhibited cell cycle progression. A reduction in the S phase was induced by DHS, whereas an increase in this phase was obtained with RSV. In addition, 50% reductions in the clonogenicity and soft agar colony formation were observed with the DHS treatment only. Finally, both stilbenes, especially DHS, reduced stemness marker expression in A549 cells and their sorted SP fraction. Spheroid formation, higher in SP cells than in the main population (MP), was significantly reduced after pretreatment with DHS, which was found to decrease SOX2 levels more than RSV. These findings indicate that stilbenes, and particularly DHS, affect stemness features of A549 cells and the SP fraction, suggesting their potential utility as anticancer agents, either alone or combined with chemotherapeutic drugs.

## 1. Introduction

Resveratrol (RSV) is a naturally occurring compound belonging to the stilbene family, which has gained extensive attention in recent years because of its potential health benefits [1,2,3,4]. Among the various resveratrol derivatives designed and synthesized with the aim of enhancing the beneficial properties of the parental molecule [5,6,7], 4,4’-dihydroxy-trans-stilbene (DHS), shown in some model systems to possess higher antioxidant [8,9,10,11] and anti-inflammatory properties [12], as well as antitumor activity [13,14,15,16,17], is one of the most promising. It shares with resveratrol several similarities, e.g., inhibiting cell proliferation by targeting specific cellular proteins, such as ribonucleotide reductase [18,19], DNA polymerase δ [20,21,22], or counteracting the expression of factors playing key roles in cancer invasion, such as MMP-2/9, N/E-cadherin, and cancer survival [17,23]. This analogue also exhibits specific characteristics that set it apart, contributing to its distinct effects. Among these, DHS demonstrates increased stability, leading to enhanced bioavailability and effectiveness, unlike resveratrol, which undergoes rapid metabolism and has a relatively short half-life [24,25,26]. Cancer remains one of the leading causes of death, as a result not only of increased incidence over the years but also because of the heterogenous responses to conventional anticancer therapies. This relates to the complexity of the tumor microenvironment, which contains, among many other cell types, a population characterized by a stem cell phenotype known as cancer stem-like cells (CSCs). These cells contribute to tumor survival and, conversely, are detrimental to patient survival [27] due to the fact that CSCs also express, in addition to a stemness gene and protein profile, ATP binding cassette (ABC) transporters, which enable them to efflux molecules and potential toxins, including chemotherapy drugs, from the cytoplasm [27]. The present study aimed to explore the antiproliferative effect of DHS and RSV at specific concentrations following a 24 h in vitro treatment on cancer cells. This was performed using the human lung adenocarcinoma cell line A549, cultured both in a monolayer and in spheroids, the latter of which allows cells to interact and better represent the actual tumor architecture and components, including CSCs [28]. In addition, a cell sorting strategy was employed to isolate the side population (SP) to enrich for CSCs. The compounds’ antiproliferative activity was analyzed by MTT assay and flow cytometry after staining with propidium iodide (PI) to assess cell cycle distribution following treatment with the stilbenes. Anchorage-dependent and independent growth was evaluated by clonogenic efficiency and soft agar assay, respectively. Finally, gene and protein expression of the stemness markers SOX2 and CD44 were investigated in 2D and 3D cultures treated with DHS and RSV for 24 h, using RT-qPCR and Western blotting, respectively.

## 2. Results

### 2.1. Cytotoxic and Antiproliferative Effects of DHS and RSV

**Figure 1:**
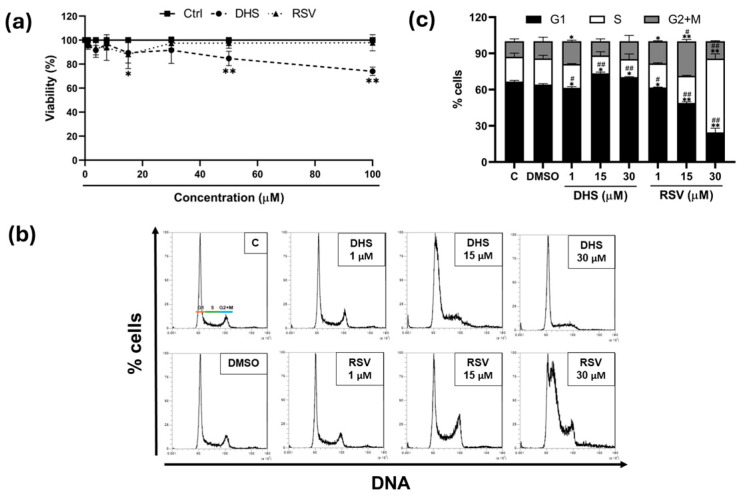
Cell viability and cell cycle profile analysis after treatment with DHS and RSV. (**a**) Cell viability was determined by MTT assay after 24 h of treatment with different concentrations of DHS or RSV (range: 1–100 μM). (**b**) Cell cycle phase distribution was evaluated by flow cytometry after staining with PI. Twenty thousand cells were analyzed from each sample, and (**c**) statistical analysis of the mean fluorescence intensity was performed with Attune NxT software v February 4, 1627.1. The results shown are the means ± SD and are representative of three independent experiments. (* *p* < 0.05 and ** *p* < 0.01 compared with control cells; # *p* < 0.05 and ## *p* < 0.01 compared with DMSO-treated cells).

The A549 cells exhibited resistance to the stilbene treatments, showing a reduction in viability only at the highest concentrations tested (Figure 1a). Statistically significant effects began at 15 µM concentration (32% reduction, *p* < 0.05), with the 30 µM dose selected as the maximum concentration for DHS in subsequent experiments (range: 1–30 μM). Representative images of the cell cycle distribution are shown in Figure 1b, with the corresponding quantitative analysis presented in Figure 1c. At the lowest concentration (1 µM), DHS induced a slight increase in the G1 phase with a corresponding reduction in the G2/M phase, while the S phase remained comparable to the DMSO vehicle control. This cell cycle alteration became more pronounced at the highest concentration (*p* < 0.01), with a marked decrease in the S-phase cell population and a significant accumulation of cells in G1. In contrast, 24 h RSV treatment resulted in a significant (*p* < 0.01) concentration-dependent increase in the S phase, compared to the control or vehicle-treated samples.

### 2.2. DHS Strongly Inhibits Both Anchorage-Dependent and -Independent Growth of A549 Cells

**Figure 2:**
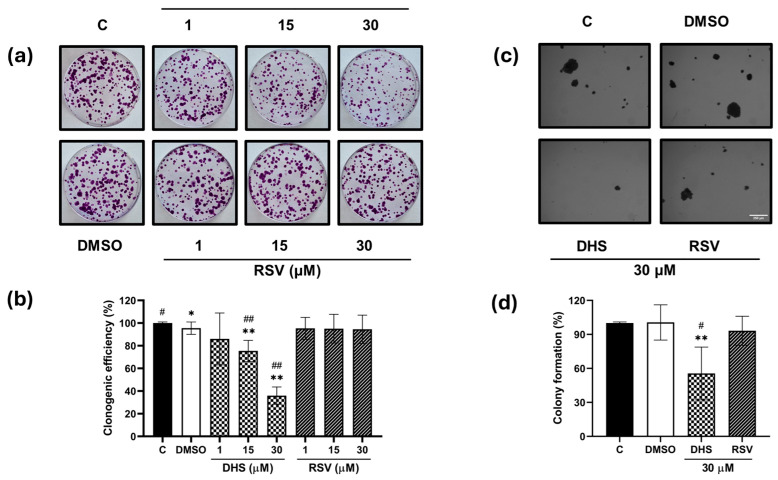
Anchorage-dependent and -independent growth of A549 cells treated with DHS or RSV. (**a**) Representative images of clonogenic efficiency of A549 cells treated with 1, 15, and 30 μM DHS or RSV for 24 h and (**b**) relative quantitative analysis. (**c**) Soft agar assay was performed treating the A549 cells with the highest concentrations of both stilbenes, and (**d**) the relative quantitative analysis. Soft agar images were obtained by stereomicroscopy at magnitude 4×, scale bar: 250 μm. The results shown are the means ± SD and are representative of three independent experiments. (* *p* < 0.05 and ** *p* < 0.01 compared with control cells; # *p* < 0.05 and ## *p* < 0.01 compared with DMSO-treated cells).

Despite the close structural similarity to RSV, DHS exhibited stronger activity. A concentration-dependent inhibition of clonogenic efficiency was observed with DHS, resulting in approximately 20, 25, and 60% reductions at increasing concentrations, compared to about a 10% reduction after RSV treatment (Figure 2a,b). Additionally, the efficacy of these compounds against the anchorage-independent growth of A549 cells, a hallmark of cancer cells, was assessed using a soft agar assay (Figure 2c,d). DHS exhibited a remarkable capacity to inhibit A549 growth and colony formation in soft agar, with a 50% reduction in the number of colonies observed at 30 μM of DHS. In contrast, no significant effect was observed in the RSV-treated cells when compared to the control or vehicle-treated samples.

### 2.3. Decrease in SOX2 and CD44 Expression after Stilbene Treatments

**Figure 3:**
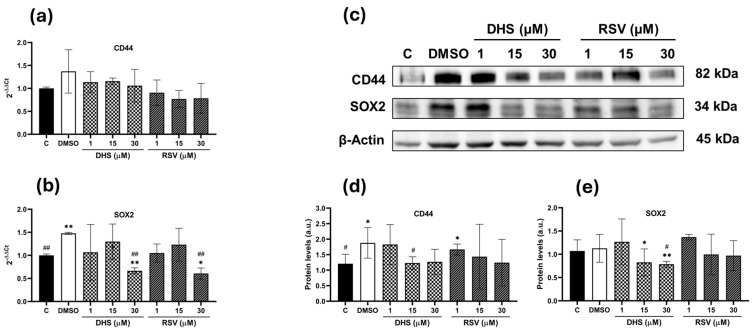
Gene and protein levels of CD44 and SOX2 in 2D A549 cells treated with 1, 15, and 30 μM DHS or RSV for 24 h. (**a**) Analysis of *CD44* and (**b**) *SOX2* transcripts by quantitative real-time PCR in A549 cells treated with stilbenes. (**c**) Representative images of CD44 and SOX2 Western blots and (**d**,**e**) the relative quantification of proteins by densitometric analysis of the Western blots and normalization to the internal loading control, β-actin. Data are the means ± SD from at least three independent experiments; values are expressed as arbitrary units (a.u.). (* *p* < 0.05 and ** *p* < 0.01 compared with control cells; # *p* < 0.05 and ## *p* < 0.01 compared with DMSO-treated cells).

The gene expression for the stemness markers *CD44* and *SOX2* was investigated in 2D cultures of A549 cells treated with 1, 15, and 30 µM DHS or RSV for 24 h. As shown in Figure 3a, a trend toward reduced *CD44* expression was observed after treatment with both stilbenes compared to the control conditions (DMSO). For *SOX2*, treatment with 30 µM DHS or RSV resulted in a statistically significant reduced expression (*p* < 0.01) compared to the untreated and DMSO controls (Figure 3b).

A similar trend was observed at the protein level, as CD44 (Figure 3c,d) showed a statistically significant reduction only after treatment with 15 µM DHS compared to the DMSO control cells. SOX2 protein levels were significantly decreased after treatment with DHS (*p* < 0.05 and *p* < 0.01) compared to both untreated and DMSO-treated cells (Figure 3e).

### 2.4. Stilbenes Impair A549 Culture as 3D Spheroids

**Figure 4:**
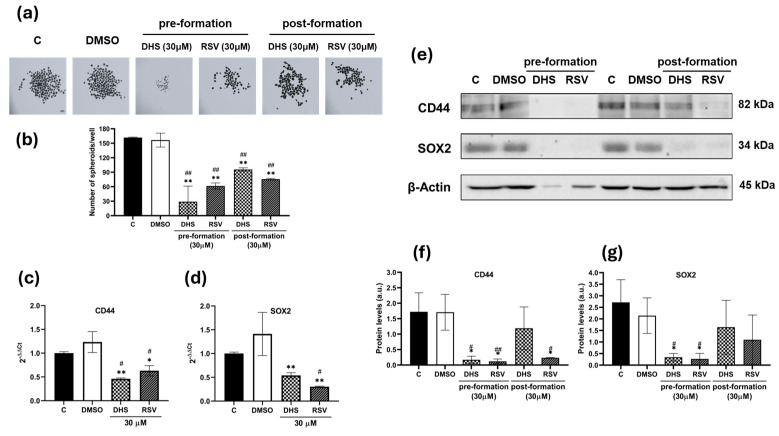
3D spheroid culture of A549 cells and their treatment with stilbenes. (**a**) Representative images of spheroids obtained in suspension and treatment with 30 μM DHS or RSV in pre- and postformation conditions of A549 cells. Images obtained by stereomicroscopy at a magnitude of 1.8×, scale bar: 250 μm. (**b**) Numbers of spheroids obtained per well under the control and treated conditions. Analysis of (**c**) *CD44* and (**d**) *SOX2* transcripts by quantitative real-time PCR in A549 spheroids treated with stilbenes. (**e**) Representative images of CD44 and SOX2 Western blots in A549 spheroids and (**f**,**g**) the relative quantification of proteins by densitometric analysis of the Western blots and normalization to the internal loading control, β-actin. Data are shown as the means ± SD from at least three independent experiments; values are expressed as arbitrary units (a.u.). (* *p* < 0.05 and ** *p* < 0.01 compared with control spheroids; # *p* < 0.05 and ## *p* < 0.01 compared with DMSO-treated spheroids).

To further probe the effect of the molecules on stemness features, A549 cells grown in 3D cultures as spheroids were treated for 24 h, with stilbenes added either at the time of cell seeding (‘preformation’) or on day 3, once spheroids were already visible in culture (‘postformation’). The resulting spheroids are shown in Figure 4a,b, with a statistically significant inhibition observed in treated cells compared to untreated and DMSO-treated cells (*p* < 0.01), particularly in the preformation condition.

Treatment with 30 µM DHS or RSV led to a significant decrease (*p* < 0.01) in *CD44* gene expression compared to the DMSO control, with a reduction in *CD44* gene expression by nearly half compared to untreated or DMSO-treated cells (Figure 4c). Similarly, the analysis of *SOX2* gene expression in 3D cultured A549 samples, as shown in Figure 4d, followed the same trend, with both stilbenes inducing a highly significant (*p* < 0.01) reduction in expression compared to the DMSO vehicle control, with DHS showing a stronger effect than RSV. At the protein levels, both stemness markers were significantly reduced in the preformation conditions after treatment with DHS or RSV (Figure 4e–g). In the postformation culture, RSV seemed to be the more effective molecule, decreasing the *CD44* expression levels in a significant manner (*p* < 0.05) in comparison to untreated and DMSO-treated cells.

### 2.5. SP Profile in A549 Tumor Cell Line

To examine the effect of the molecules on the stem cell fraction within the A549 cells, Hoechst 33342^low/neg^ cells (SP fraction) and Hoechst 33342^high/pos^ cells (MP fraction) were sorted from A549 cells by flow cytometry (Figure S1).

**Figure 5:**
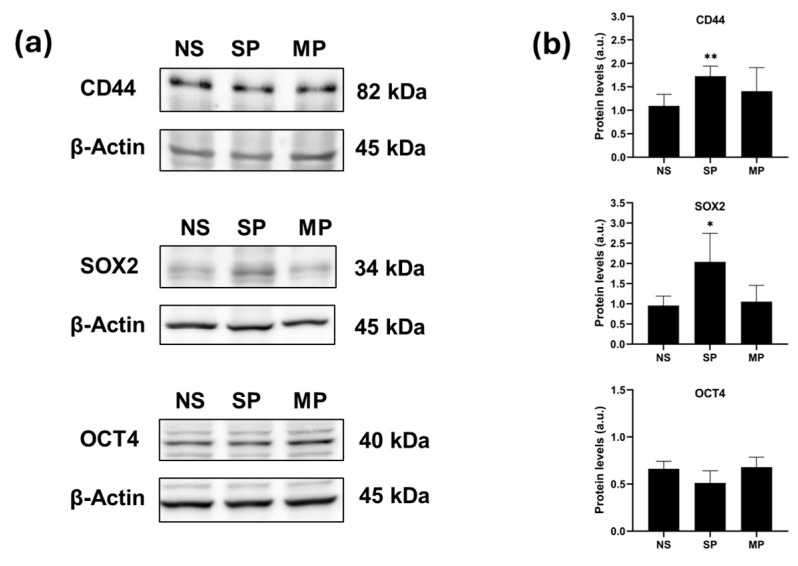
Characterization of SP fraction from A549 cells by Western blot analyses of stemness markers CD44, SOX2, and OCT4. (**a**) Representative images of CD44, SOX2, and OCT4 Western blots in A549 sorted cells (SP and MP) and non-sorted cells (NS). (**b**) Relative quantification of the proteins by densitometric analysis of the Western blots and normalization to the internal loading control, β-actin. Data are shown as the means ± SD from at least three independent experiments; values are expressed as arbitrary units (a.u.). (* *p* < 0.05 and ** *p* < 0.01 compared with NS cells).

To further explore the differences between SP, MP and non-sorted (NS) cells, Western blot analyses were performed for the stemness markers CD44, OCT4, and SOX2 (Figure 5a,b). SP cells showed higher expression levels of CD44 and SOX2 in comparison to MP and NS cells, while no significant differences in levels of OCT4 were observed.

### 2.6. DHS Decreases Anchorage-Independent Growth and SOX2 Expression Postsorting

To evaluate the effect of stilbene treatment on SP and MP, both populations were treated with 30 μM of either molecule and subjected to a soft agar colony formation assay (Figure S2). The number of colonies obtained was significantly reduced following treatment with DHS for both the SP and MP subpopulations, while RSV treatment resulted in a decrease in growth primarily in the SP subpopulation, and only in comparison with untreated cells.

**Figure 6:**
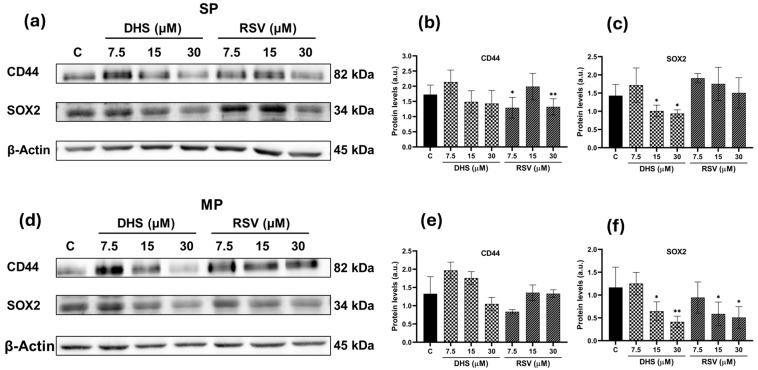
Protein levels of CD44 and SOX2 in SP and MP cells after 7.5, 15, and 30 μM RSV or DHS for 24 h of treatment. (**a**) Representative images of CD44 and SOX2 Western blots in SP cells and (**b**,**c**) the relative quantification of proteins by densitometric analysis of the Western blots and normalization to the internal loading control, β-actin. (**d**) Representative images of CD44 and SOX2 Western blots in MP cells and relative quantification of proteins by densitometric analysis of the Western blots and normalization to the internal loading control, β-actin (**e**,**f**). Data are shown as the means ± SD from at least three independent experiments; values are expressed as arbitrary units (a.u.). (* *p* < 0.05 and ** *p* < 0.01 compared with control cells).

In parallel, SP and MP cells were exposed to increasing concentrations (7.5, 15, and 30 µM) of DHS or RSV for 24 h. Figure 6a shows CD44 and SOX2 protein levels in SP cells, with relative quantitative analysis provided in Figure 6b,c. SOX2 seems to be more sensitive to DHS treatment, demonstrating a 30–40% reduction in SP cells. A trend towards reduction was observed for CD44 protein levels in SP subpopulations after treatment with DHS, whereas RSV treatment induced significant reductions at 7.5 and 30 µM concentrations (*p* < 0.05 and *p* < 0.01). In the MP subpopulation (Figure 6d–f), the decrease in SOX2 expression levels was even more significant (*p* < 0.05 and *p* < 0.01) for both stilbenes treatments. A trend towards reduction was observed for CD44 protein levels both in MP subpopulations after treatment with DHS or RSV, although the data did not reach statistical significance.

### 2.7. Stilbenes on A549 Spheroids Postsorting

**Figure 7:**
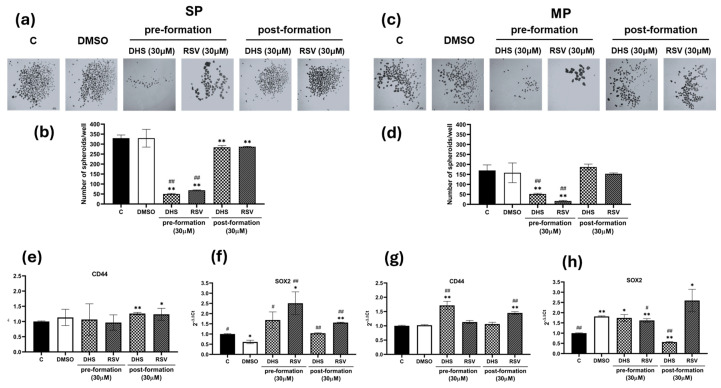
3D culture of sorted A549 cells (SP and MP) and their treatment with stilbenes. (**a**) Representative images of spheroids obtained in suspension and treatment with 30 μM DHS or RSV in pre- and postformation conditions of SP cells. (**b**) Numbers of spheroids obtained per well in the control and treated conditions. (**c**) Representative images of spheroids obtained in suspension and treatment with 30 μM DHS or RSV in pre- and postformation conditions of MP cells. (**d**) Numbers of spheroids obtained in each well in control and treated conditions. (**e**) Analysis of *CD44*, (**f**) *SOX2* transcripts by quantitative real-time PCR in SP spheroids treated with stilbenes. (**g**) Analysis of *CD44*, (**h**) *SOX2* transcripts by quantitative real-time PCR in MP spheroids treated with stilbenes. Images were obtained by stereomicroscopy at a magnitude of 1.8×, scale bar: 250 μm. Data are shown as the means ± SD from at least three independent experiments; values are expressed as arbitrary units (a.u.). (* *p* < 0.05 and ** *p* < 0.01 compared with control spheroids; # *p* < 0.05 and ## *p* < 0.01 compared with DMSO-treated spheroids).

The effect of stilbenes on tumorsphere growth from SP and MP cells was analyzed by incubating both fractions in the presence or absence of stilbenes (30 μM). The following two modalities were used in parallel: one where the treatments were added immediately after cell seeding (preformation) and another where the molecules were added only after spheroid formation on day 3 (postformation). After 10 days of culture, the morphology and quantity of spheroids were evaluated by microscopy, as shown in Figure 7a,c. The cytotoxic effects of DHS on spheroids were clearly evident for both subpopulations, in particular in the pretreatment condition. The spheroids derived from MP cells were less proliferative in comparison to those from SP cells (Figure 7b,d), and DHS significantly inhibited spheroid growth in both fractions (*p* < 0.01) compared to untreated or DMSO control cells. In SP spheroids, the gene expression levels of *CD44* and *SOX2* (Figure 7e,f), showed a significant increase, particularly in SOX2 levels, after treatment with both stilbenes in pre- and postformation cultures (*p* < 0.05 and *p* < 0.01) in comparison to untreated or DMSO-treated cells. A similar behavior was observed in MP spheroids for *SOX2* and *CD44* gene expression levels (Figure 7g,h), with only 30 µM DHS in postformation conditions inducing a statistically significant decrease (*p* < 0.01).

**Figure 8:**
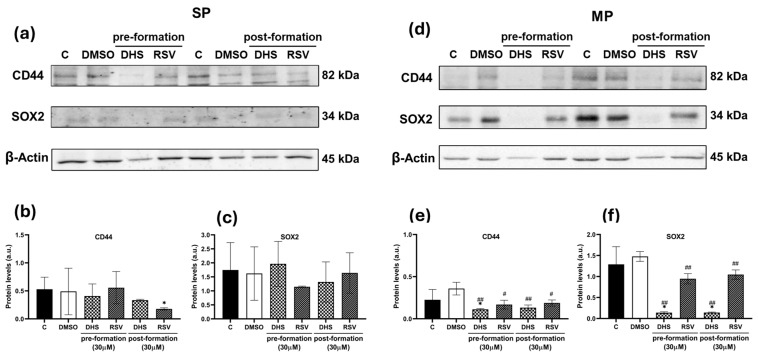
Protein levels of CD44 and SOX2 in SP and MP spheroids after 30 μM DHS or RSV in pre- and postformation conditions. (**a**) Representative images of CD44 and SOX2 Western blots in SP spheroids and (**b**,**c**) the relative quantification of proteins by densitometric analysis of the Western blots and normalization to the internal loading control, β-actin. (**d**) Representative images of CD44 and SOX2 Western blots in MP spheroids and (**e**,**f**) the relative quantification of proteins by densitometric analysis of the Western blots and normalization to the internal loading control, β-actin. Data are shown as the means ± SD from at least three independent experiments; values are expressed as arbitrary units (a.u.). (* *p* < 0.05 compared with control spheroids; # *p* < 0.05 and ## *p* < 0.01 compared with DMSO-treated spheroids).

In SP spheroids, only 30 µM RSV was able to significantly decrease CD44 protein levels in the postformation culture in comparison to untreated cells (Figure 8a,b), whereas SOX2 protein levels remained similar to those in untreated and DMSO-treated cells (Figure 8a,c). In MP spheroids, both CD44 and SOX2 protein levels were significantly reduced after treatment with both stilbenes in pre- and postformation culture (Figure 8d–f).

## 3. Discussion

The present study investigated the effects of RSV and its analogue DHS on A549 cells, with a specific focus on stemness features. RSV has been observed to induce differentiation of different cell types such as 3T3-L1 pre-adipocytes [29], and to exert pleiotropic effects on various signaling pathways involved in maintaining stemness [30]. However, the impact of the RSV analogue DHS on CSCs has not been explored until now. The use of human lung adenocarcinoma A549 aimed to compare the effects of DHS on stemness in comparison to those of RSV.

A549 cells appeared to be resistant to stilbene treatments, with no significant reduction in cell viability observed for RSV concentration up to 100 μM, while DHS showed a reduction in viability starting at 15 µM, although the cytotoxicity threshold remained above 70% [10]. This is in line with previous structure/function studies performed on different cell types, where DHS showed a stronger effect than resveratrol, possibly due to the position of the OH in 4 and 4’f facilitating the interaction with molecules involved in cell proliferation, such as polymerases [10]. DHS-treated A549 cells demonstrated a reduction in the number of S-phase cells, with a concomitant increase in G1-phase cells, whereas RSV-treated samples showed a delay in the onset of the S phase [13,22]. A similar S-phase arrest has been reported for A549 cells exposed to RSV treatment, in a dose-dependent manner up to 150 μM, which led to an increased rate of apoptosis [31]. However, in the present study, no significant induction of apoptosis was observed for either DHS or RSV up to 30 μM, as confirmed by the lack of the sub-G1 peak and the absence of morphological apoptotic features. These results are in line with previous data obtained in our laboratory in other cancer cell lines [13,14].

A strong inhibition in both anchorage-dependent and -independent growth was observed after 30 μM DHS treatment, accompanied by a reduction in CSC markers SOX2 and CD44 at both the gene and protein levels, with DHS being more potent than RSV. This observation was extended to a 3D spheroid culture model, selected for its ability to better mimic tumor architecture and resistance to chemotherapy compared to monolayer models [32]. Both DHS and RSV were able to significantly hamper spheroid growth in pre- and postformation models, simulating chemopreventive and chemotherapeutic treatments, respectively. Even more clearly than in the 2D model, stilbene treatment further reduced CD44 and SOX2 expression in spheroids, regardless of whether the molecules were added pre- or postformation. Improving cancer chemosensitivity while minimizing undesirable side effects is critical to improve quality of life and therapeutic outcomes for cancer patients. Recently, Mohammadhosseinpour et al. demonstrated that the prenylated stilbenoid arachidin-1 (A-1) enhanced the anticancer effects of Paclitaxel (Pac) in triple-negative breast cancer (TNBC) cells [33]. A-1 and Pac combined treatment inhibited TNBC spheroid growth through cell proliferation inhibition, apoptosis induction mediated by mitochondrial oxidative stress, whereas in the same experimental model, RSV appeared to be the least active molecule. The present study suggests that DHS may hold similar promise in combination therapies, given its superior performance in reducing spheroid growth and stemness marker expression compared to RSV.

Considering the role of stem cells in tumor formation and resistance, A549 were subsequently sorted on the basis of the Side Population, using FACS technology and Hoechst 33342 dye [34], a technique widely used to enrich for CSCs because of the activity of ATP-binding cassette (ABC) transporters [35]. This mechanism is responsible for chemoresistance in both normal and tumor stem cells [36] and has been exploited in a number of cell models including A549 cells [37,38,39,40]. The sorting of A549 cells showed that 1.4% of cells were mildly positive for Hoechst 33342 dye, indicative of the SP phenotype, which was reduced in the presence of Verapamil, an inhibitor of ABC subfamily efflux pumps. This result is in line with what has been reported in the literature, although the proportion of the SP fraction appears low in comparison to other published studies carried out on A549, which vary between 2 and 24% [41,42,43]. Molecular assays after sorting confirmed a higher expression of the CSC markers SOX2 and CD44 in SP cells compared to both MP and non-sorted (NS) A549 cells used as control. SP cells also exhibited a greater capacity for spheroid formation in 3D cultures, indicating a higher stemness potential.

In addition, in a soft agar colony formation assay, which is indicative of tumorigenic phenotype [41], all the three cell populations demonstrated the ability to grow independently on semi-solid medium, with SP cells being the most efficient. This malignancy behavior aligns with previous findings that SP cells from A549 cells exhibit enhanced tumorigenic potential [41]. Treatment with 30 μM DHS or RSV reduced colony formation in SP cells, indicating that stilbene treatment can alter the anchorage-independent growth of A549 SP cells. When applied to spheroid cultures, both RSV and DHS significantly reduced spheroid production in SP and MP fractions, particularly in the preformation condition. The inhibitory effect was less pronounced in the postformation phase, reaching a significance for only SP cells, possibly due to the shorter treatment duration (stilbenes present for 7 days) in the postformation compared to the preformation conditions (10 days). Recently, Wang et al. demonstrated that inhalable resveratrol-cyclodextrin complex loaded biodegradable nanoparticles enhanced efficacy against non-small cell lung cancer. In their work the authors highlighted an intensified anticancer effect of these RSV-containing nanoparticles in a 3D spheroid model [43]. In addition, Shankar and colleagues [44] reported that RSV was able to inhibit the formation of spheroids in pancreatic cancer CSCs after 7 days of treatment at concentrations of 0, 10, 20, and 30 µM. Barros and colleagues [45] also showed that RSV, when combined with doxorubicin, exhibited promising antitumor activity against breast, cervical and liver cancer, while Esposito et al. [46] reported the effect of RSV on the growth of spheroids derived from ovarian tumor cells at concentrations of 10, 20, and 100 µM. Given the pronounced effect of DHS observed in the present study, which outperformed RSV in the A549 spheroid model, it would be of significant interest to assess the performance of this compound in other cancer spheroid models and compare its effects with those reported in these previous studies. In addition, extending the treatment duration beyond 24 h could provide complementary information regarding the longer term effects of the stilbenes on these cancer traits.

Since stemness characteristics are based on the expression of specific markers, such as SOX2 and CD44, their protein levels, both in 2D and 3D cell cultures treated with stilbenes, were evaluated by Western blotting. SOX2 levels, which were highest in SP cells, appeared to be the most affected by treatment with stilbenes. In fact, in SP, but also MP and NS cells, its levels were reduced in a concentration-dependent manner after treatment with DHS. Additionally, in NS and MP spheroids, a reduction in CD44 and SOX2 markers after treatment with either DHS or RSV was observed. Surprisingly, in SP spheroids, the changes in these markers did not reach statistical significance, which aligns with the data obtained by RT-qPCR, suggesting the need for further analysis.

It is reported in the literature that RSV can exert inhibitory effects on the growth of CSCs of different tumor types, both independently and in synergy with other compounds [47]. Furthermore, preclinical studies in animal and clinical models have demonstrated that RSV, when combined with standard chemotherapy drugs, enhances therapeutic efficacy by sensitizing tumor cells to the effects of chemotherapeutics and overcoming multi-drug resistance, although some side effects, such as diarrhea, nausea, and loss of appetite, have been reported in humans [48]. In light of these findings, along with the potent response observed for DHS at 30 μM in this study compared to RSV, further preclinical investigations to evaluate the potential translational benefits of DHS, either as a standalone treatment or as an adjuvant in future anticancer therapy trials.

## 4. Materials and Methods

### 4.1. Reagents and Cell Culture 

4,4’-Dihydroxy-trans-stilbene (DHS) and resveratrol (RSV) were purchased from Santa Cruz Biotechnology (Dallas, TX, USA) and dissolved in dimethyl sulfoxide (DMSO) to a concentration of 200 mM, subsequently stored at −20°C. For treatment, medium was supplemented with different concentration of stilbenes as described. A549, lung adenocarcinoma cell line (kindly provided by Dr. L. Iamele), was cultured in DMEM supplemented with 10% fetal bovine serum (FBS), 200 mM L-glutamine, 100 IU/mL penicillin, and 100 µg/mL streptomycin, all obtained from Thermo Fisher Scientific (Waltham, MA, USA), in a 37 °C humidified incubator under atmospheric conditions with supplementation of 5% CO_2_. Verapamil hydrochloride was provided by Thermo Fisher Scientific, solubilized in EtOH 100% to obtain a 100 mM stock solution and used in the 25 μM working solution for cytometric analysis.

For 3D spheroid cultures, cells were incubated with agitation onto a shaker set at 150 rpm. Cells were seeded at a density of 1.2 × 10^6^ cells per well in a 6-well plate, in the following two experimental conditions: pre- or postspheroids formation. In particular, in the preformation conditions the cells were treated with 30 µM DHS or RSV immediately with the seeding; for the postformation condition, 72 h after the cells seeding, spheroids were treated with both stilbenes up to 10 days, changing with medium every three days. 

### 4.2. Isolation of SP Cells from A549 by Sorting

To identify and characterize CSCs in the A549 lung adenocarcinoma cell line, SP cells were isolated by fluorescence-activated cell sorting (FACS) as previously described [34]. Cells were labeled with 5 µg/mL Hoechst 33342 (Sigma Aldrich, St. Louis, IL, USA) for 90 min at 37 °C. The Hoechst dye was excited with UV laser at 405 nm and its fluorescence was measured with 450/40 and 660/20 long pass filter. SP cells that expressed ABC transporters (ABCG2) and demonstrated Hoechst 33342 efflux activity were sorted by FACSaria III (Becton Dickinson, Temecula, CA, USA) flow cytometry (Centro Grandi Strumenti, CGS, University of Pavia, Pavia, Italy). Untreated control cells were incubated in the presence of 50 µM Verapamil (Thermo Fisher Scientific), a known ABC transporter inhibitor.

### 4.3. Cytotoxicity and Cell Cycle

Cell toxicity was determined by the MTT [3-(4,5-dimethylthiazol-2-yl)-2,5-diphenyltetrazolium bromide] assay. A549 cells, seeded at a density of 1 × 10^4^ in 96-well plate, were treated for 24 h with 1, 3.75, 7.5, 15, 30, 50, and 100 µM of either DHS or RSV, then processed as previously describe [13]. 

For the analysis of DNA content, A549 were treated for 24 h with 1, 15, and 30 µM of DHS or RSV. At the end of the incubation period, cells were trypsinized, washed in PBS, and resuspended in PBS containing 100 µg/mL RNase A, 0.05% NP-40 and 5 µg/mL propidium iodide (PI). Staining occurred for at least 30 min at room temperature (RT). Twenty thousand cells were analyzed for each sample with a BD FACS Lyric cytometer (CGS, University of Pavia), and the data were analyzed using the Attune NxT software v February 4, 1627.1.

### 4.4. Clonogenic Efficiency and Soft Agar Assays

The clonogenic efficiency was determined after incubation of cells in culture medium containing DHS and RSV. Briefly, the cells were trypsinized, diluted in complete DMEM medium and seeded at 200 cells per 60 mm dish, in triplicates. After 24 h of treatment with stilbenes, the cells were washed twice with PBS and 5 mL of fresh medium was added. After 10 days, the colonies were stained with crystal violet and counted, and the clonogenic efficiency was calculated as the mean percentage with respect to control cells. To test anchorage-independent growth, A549 cells were trypsinized and resuspended to obtain a density of 2 × 10^4^ cells in 500 μL of DMEM containing 20% FBS. DHS or RSV (30 μM) was added directly to the cell suspension together with 500 μL of 0.33% Bacto Agar (Difco Laboratories, Franklin Lakes, NJ, USA). The mixtures were poured quickly in culture cell dishes prepared in advance with 5 mL of 0.6% Bacto agar in Dulbecco’s modified Eagle’s medium containing 10% FBS and incubated at 37 °C for 3 weeks. The colonies formed were photographed and counted using a ×20 magnification inverted microscope (Leitz DM-IL, Leica, Wetzlar, Germany).

### 4.5. Western Blotting and Antibodies

Cells were treated with different concentrations of DHS or RSV as indicated in the results. Proteins related to stemness were investigated by Western blotting, as described previously [22], using the following primary antibodies: anti-CD44 (Invitrogen, Waltham, MA, USA, 8E2F3, 1:1000), anti-β-Actin (Invitrogen, MA5-15739 1:5000), anti-SOX2 (Abcam, Cambridge, UK, ab97959 1:1000), anti-Oct4 (Abcam, ab19857, 1:1000). Densitometric analyses were performed with the Fiji software [49].

### 4.6. Quantitative Real-Time RT-qPCR

The A549 cells were treated with DHS or RSV at 1, 15, and 30 µM concentrations for 24 h. For 3D experiments, SP and MP spheroids were exposed to a chronic treatment with 30 µM of either stilbene. Total RNA was extracted from cultured cells using TRIzol™ Reagent (Thermo Fisher Scientific) according to the manufacturer’s instructions. The concentration was measured on a Nanodrop spectrophotometer (DeNovix, Wilmington, DE, USA), and samples were diluted to a final concentration of 1000 ng/µL. For each sample, 1 µg RNA was reverse transcribed using the specific High Capacity cDNA Reverse Transcription kit (Applied Biosystems, Waltham, MA, USA), following manufacturer’s recommendation.

For RT-qPCR expression profiling, 1 µL of each cDNA sample was amplified using SYBR™ Green PCR Master Mix (2X) (Applied Biosystems) for human *CD44* (forward primer 5’-ACACACGAAGGAAAGCAGGA-3’, reverse primer 5’-CACTGGGGTGGAATGTGTCT-3’) and *SOX2* (Forward primer 5’-TTTGTCGGAGACGGAGAAGC-3’, reverse primer 5’-CCCGCTCGCCATGCTATT-3’) in 20 µL reactions. Glyceraldehyde-3-phosphate dehydrogenase (*GAPDH*, forward primer 5’-TGGTATCGTGGAAGGACTCATGAC-3’, reverse primer 5’-ATGCCAGTGAGCTTCCCGTTCAGC-3’) was used as internal reference and co-amplified with target samples using identical experimental conditions. Real-time fluorescence monitoring was performed with the CFX Connect Real-Time PCR System (Bio-Rad, Hercules, CA, USA). Reactions were performed in triplicate.

### 4.7. Statistical Analysis

At least three biological replicates (unless otherwise stated) were performed for each experiment. Statistical analysis was carried out to calculate significance with the Student *t*-test (two-tailed), with *p*-values < 0.05 considered to be significant.

## Data Availability

Dataset available upon reasonable request from the authors.

## Supplementary Materials

The following supporting information can be downloaded at: https://www.mdpi.com/article/10.3390/ijms251910390/s1.
