# Supplementary material for: Stilbene Treatment Reduces Stemness Features in Human Lung Adenocarcinoma Model
Source: Int J Mol Sci. 2024 Sep 27;25(19):10390. doi: 10.3390/ijms251910390 (PMC11476666; doi:10.3390/ijms251910390)
Supplement: Supplementary file 1 [file ijms-25-10390-s001.zip › ijms-3170375-supplementary.pdf]

Figure S1

(a)

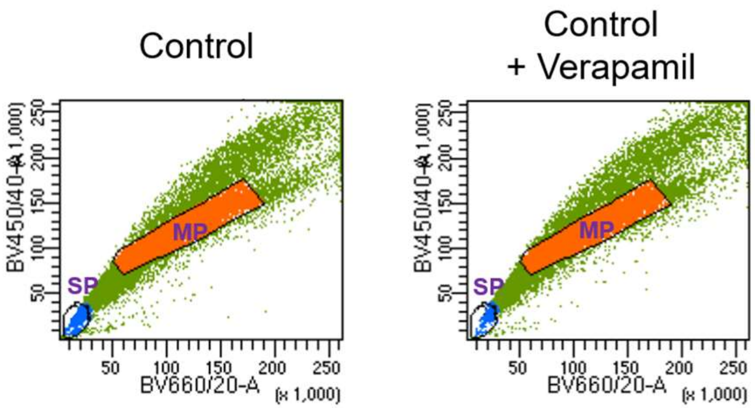

Subpopulations	Number of cells post sorting	Fluorescence (%) of control cells	Fluorescence (%) of cells with Verapamil
Side Population	11˙000	1.4%	0.7%
Main Population	526˙000	34.6%	37.4%

The Hoechst 33342$^{low/neg}$ fraction accounted for 1.4% of live cells and the percentage decreased to 0.7% subsequent to treatment with Verapamil (Figure S1a). SP and MP cells were put in culture and after 4 days no notable morphological difference was observed between the two subpopulations in comparison to the non-sorted cells (NS) (Figure S1b).

(b)

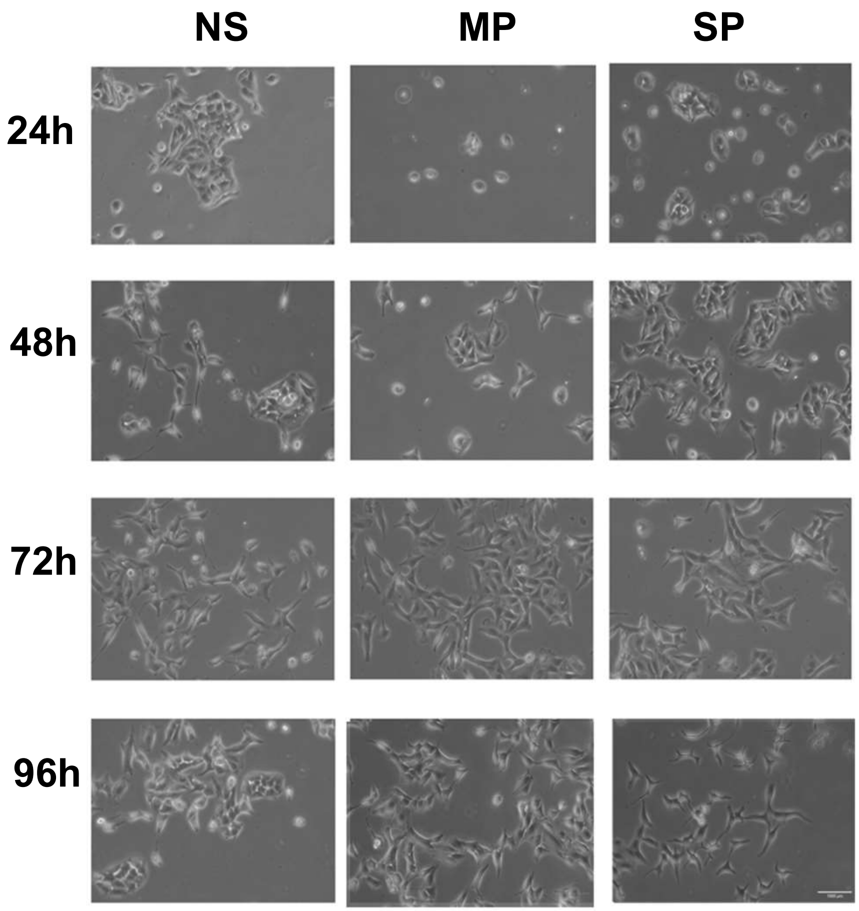

Figure S1

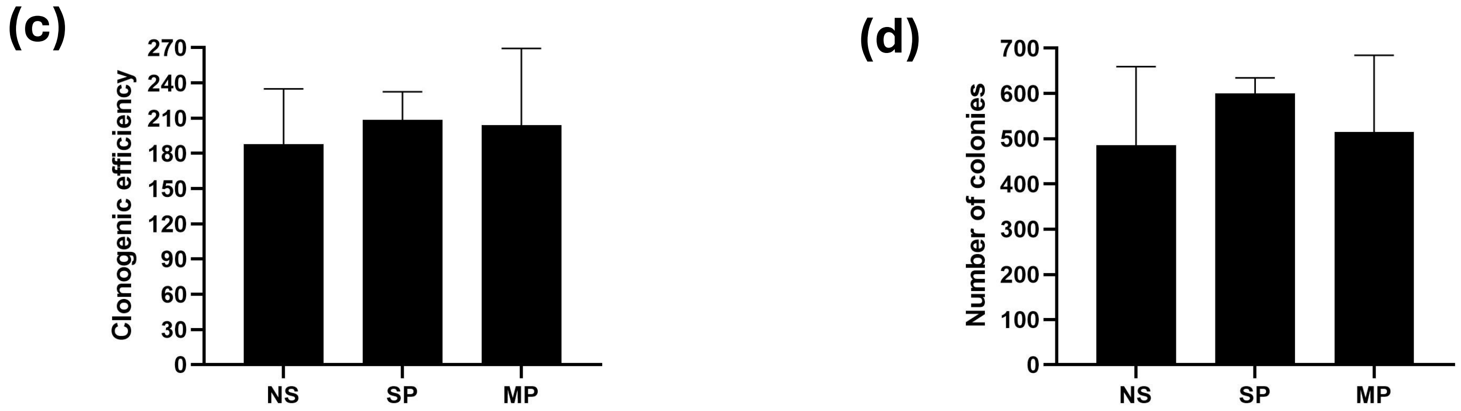

A clonogenic assay performed to analyze the tumorigenicity of SP and MP cells in vitro (Figure S1c) indicated no differences between SP and MP clonogenic efficiency in comparison to NS cells. A similar behaviour was evident for anchorage-independent growth analysed in soft agar (Figure S1d).

Figure S2

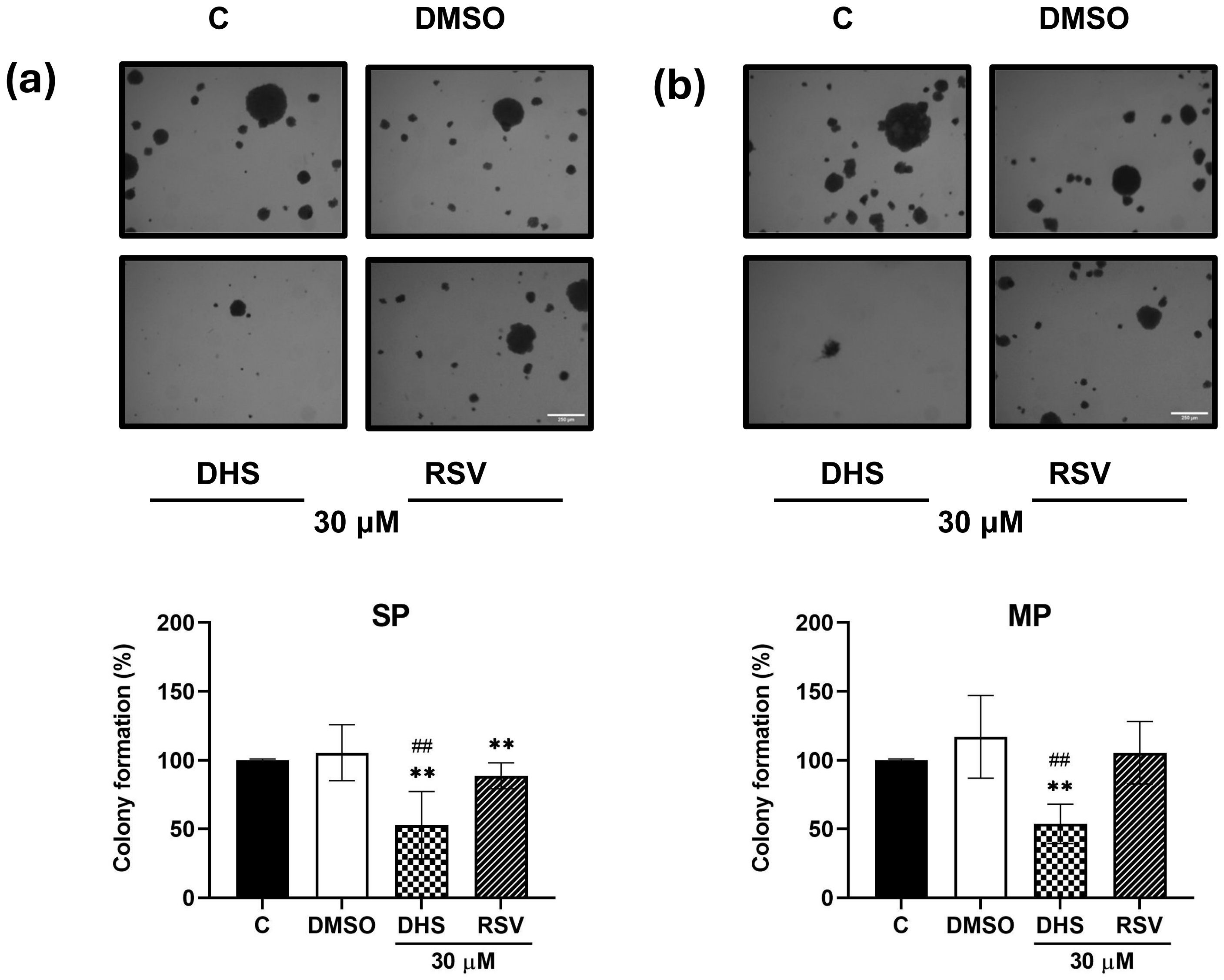

Soft agar assay of SP (a) and MP (b) populations treated with 30 μM of either molecule and quantification of colony formation
